# Introducing *Annals of Surgical Innovation and Research*

**DOI:** 10.1186/1750-1164-1-1

**Published:** 2007-02-20

**Authors:** Ronald Matteotti, Michel Gagner, James Becker

**Affiliations:** 1 Annals Of Surgical Innovation and Research, BioMed Central Ltd, Middlesex House, 34-42 Cleveland Street, London W1T 4LB, UK; 2 University Hospitals of Cleveland, Case Western Reserve University, Cleveland, OH, 44106, USA; 3 New York Presbyterian Hospital, Weill-Cornell Medical Center, New York, NY,10021, USA; 4 Boston University Medical Center, Boston, MA 02118, USA

We are proud to introduce *Annals of Surgical Innovation and Research *[1], a new forum for cutting-edge findings and innovations in all surgical specialties. This journal is entirely open access, peer-reviewed and online. We are interested in articles from all areas of surgery, minimal invasive techniques and endoscopy, and related bioengineering. We want to promote the exchange of ideas, concepts and findings across all surgical specialties. The following types of articles will be published: research, short communications, hypotheses, commentaries, reviews (as invited articles), case reports, case studies, meeting reports, letters to the editor and book reviews.

Open access provides an unparalleled opportunity to present information to specialists and the public. The online appearance of *Annals of Surgical Innovation and Research *(ASIR) allows the immediate publication of accepted articles and the presentation of large amounts of data, multimedia files and supplemental information.

But do we really need another journal in the field of medicine?

Yes we do, because this open access journal will be accessible to everyone, and not just those with access to a library with a subscription. As such, ASIR will help to increase public interest in, and support of, research. Many major founders, such as the NIH and the Wellcome Trust, now have policies to encourage grant holders to release their research to the public as soon as possible after publication [2-4]. These policies are likely to be replicated by other major founders, especially following the recommendations of the UK House of Commons Committee [5].

A country's economy will not influence its researchers' ability to access articles in ASIR, because resource-poor countries (and institutions) will be able to read the same material as wealthier ones [6]).

Our ambitious aim is to function as an ideal bridge to close the gap between the revolution in surgical innovations and research, especially in minimal invasive surgical technology, and to help surgeons at the front line of patient care. The journal tries to centralize new knowledge in one forum to give a quick and comprehensive review over new innovations and relevant research. The scope of the journal is to advance the quickly developing field of new surgical techniques and innovations in a more and more technically demanding field. It offers an opportunity to publish large datasets, large numbers of illustrations and even movies, and therefore to act as the primary source for surgical innovations to be used by clinicians and researchers.

We recognize the need for a dynamic forum that fosters the exchange of ideas across the entire surgical community, a journal that is open access and online. We now have an opportunity to make *Annals of Surgical Innovation and Research *just such a journal, and this is an exciting challenge. Assisting me in this new venture is an editorial board, which consist of members out of 14 different countries, many of them true leaders, others very enthusiastic and soon to be.

For *Annals of Surgical Innovation and Research *to become an exciting forum for surgical research and innovation, we need you to contribute your most innovative and significant work in your field. In exchange, *Annals of Surgical Innovation and Research *will provide a fast, fair, and constructive review process; the highest quality production values; and a dedicated team of professional scientific editors, who are willing to discuss your research. All manuscripts are reviewed on the basis of scientific merit and held to the highest standards of excellence and editorial consistency. Upon acceptance they will be immediately available in PubMed and indexed by several other services.

I would like to take this opportunity to acknowledge our Editorial Board [7] for their commitment to the journal and to express my most sincere gratitude to the entire staff of BioMed Central [8] for their valuable advice and support in launching this journal. Special thanks to Christian Schmidt, Deputy Editor of *Molecular Cancer *[9], who guided me through the initial steps of this adventure.
